# Commercially purchased and in-house bred C57BL/6 mice with different gut microbiota exhibit distinct indomethacin-induced toxicities

**DOI:** 10.1080/29933935.2025.2585683

**Published:** 2025-12-07

**Authors:** Jianan Zhang, Rose Viguna Thomas Backet, Josh J. Sekela, Meredith J. Zeller, Rani S. Sellers, Matthew R. Redinbo, Ajay S. Gulati, Aadra P. Bhatt

**Affiliations:** a Department of Chemistry, University of North Carolina at Chapel Hill, Chapel Hill, NC, USA; b Department of Pediatrics, Division of Gastroenterology, University of North Carolina at Chapel Hill, Chapel Hill, NC, USA; c Division of Gastroenterology and Hepatology, Department of Medicine, Center for Gastrointestinal Biology and Disease, University of North Carolina at Chapel Hill, Chapel Hill, NC, USA; d Department of Pathology and Laboratory Medicine, University of North Carolina at Chapel Hill, Chapel Hill, NC, USA; e Departments of Biochemistry and Biophysics, Microbiology and Immunology, and Integrated Program in Biological and Genome Sciences, University of North Carolina at Chapel Hill, Chapel Hill, NC, USA

**Keywords:** NSAID, intestinal toxicity, gut microbiota, microbial enzymes, β-glucuronidase, mucolytic enzymes, vendor differences

## Abstract

Nonsteroidal anti-inflammatory drug (NSAID)-induced toxicities are a significant clinical problem, yet the factors influencing these outcomes remain incompletely understood. Here, we investigated the impact of a mouse vendor on indomethacin-induced injury using C57BL/6 mice from different breeding facilities (in-house ‘Tar Heel’ and commercial Charles River). We found that Tar Heel mice exhibited significantly greater susceptibility to indomethacin toxicity, characterized by greater body weight loss, increased ileal ulceration, elevated fecal lipocalin-2 levels, and higher goblet cell numbers in the ileum compared to Charles River mice. Importantly, whole-genome metagenomic analysis revealed distinct baseline gut microbiomes between the two types of mice. Notably, Tar Heel mice presented increased abundances of *β*-glucuronidase (GUS)-producing bacteria, particularly those expressing Loop−1 GUS enzymes, and elevated levels of mucolytic enzyme-encoding bacteria. These differences suggest that the increased indomethacin toxicity observed in Tar Heel mice may be related to functional changes in their gut microbiome, which may predispose them to an exaggerated response to NSAID exposure. Together, our findings demonstrate that vendor-specific differences significantly influence NSAID-induced intestinal toxicity and highlight the importance of considering mouse sources and gut microbial compositions in experimental design. Moreover, we highlight the potential functional roles that gut microbes play in host–indomethacin interactions.

## Introduction

Gastrointestinal (GI) toxicity is a significant side effect of nonsteroidal anti-inflammatory drugs (NSAIDs), such as indomethacin, which are utilized by millions of patients worldwide. As many as 16,500 deaths annually in the US are caused by NSAID-induced GI bleeding.^1-3^ In addition to the well-described gastric damage, NSAIDs also cause enteropathy resulting from the interactions between host physiology and the gut microbiome, as well as the biochemical properties of the NSAIDs themselves.^4-6^ NSAIDs are amphiphilic molecules whose physicochemical properties allow them to penetrate through both the intestinal mucus layer and phospholipid-rich epithelial cell membranes. NSAIDs can result in increased mucosal exposure to luminal aggressors such as gut bacteria. At physiologically relevant concentrations, NSAIDs are known to cause mitochondrial uncoupling, leading to increased death of intestinal stem and differentiated cells and increased intestinal permeability.^7^^,^^8^

The gut microbiota has an additional role in NSAID-induced toxicity. A range of NSAIDs, including indomethacin, are conjugated with glucuronic acid in the liver and excreted into the intestine as inactive glucuronide metabolites. Many intestinal bacterial species produce *β*-glucuronidase (GUS) enzymes that deconjugate these metabolites, thereby reactivating the drug and increasing local gut luminal concentrations, which further contribute to intestinal damage.^9^ The abundance and activity of GUS-producing bacteria in the GI tract can therefore significantly impact the severity of NSAID-induced intestinal damage.^10^^,^^11^ Furthermore, host factors such as differential cyclooxygenase (COX) activities, responses to redox stress, innate immunity, and the induction of inflammatory cytokines lead to further epithelial erosion and ulceration.^12^

The gut microbiota functionally influences the toxicity profile of NSAIDs in the host. Intestinal mucus, which is produced primarily by goblet cells, forms a crucial protective barrier against luminal contents and drug-induced injury. Some bacterial species produce mucolytic enzymes that can degrade this protective layer.^13^ In the context of NSAID use, an overabundance of these mucolytic bacteria may compromise the mucus barrier, exacerbating drug-induced damage to the underlying epithelium. Moreover, these microbial factors interact with the host, particularly with structures such as goblet cells, which are essential for maintaining intestinal homeostasis.^14^ NSAID-induced alterations in goblet cell function or number,^15^ potentially influenced by the local microbial environment, could further contribute to gut toxicity.

Animal models, particularly mice, are invaluable tools for investigating NSAID-induced GI toxicity and potential therapeutic interventions.^16^^,^^17^ These models facilitate the study of mechanisms involved in drug-induced damage and can test novel protective strategies under controlled conditions. However, the source of laboratory animals – whether they are bred in-house or obtained from commercial suppliers – may introduce variability in experimental outcomes. Advances in metagenomic sequencing technologies have revolutionized our ability to characterize complex microbial communities with unprecedented depth and resolution. Whereas previous studies have shown that differential microbiota composition between vendors is an important consideration in rodents,^18^^,^^19^ the mechanisms driving these differences remain unclear.

Understanding the interplay between the function of the gut microbiome, its enzymatic activities, and host cellular responses is crucial for elucidating the mechanisms of NSAID-induced GI toxicity, with the goal of preventing or mitigating this damage. Moreover, differences in the abundance or activity of these key microbial players between in-house bred and commercially sourced mice could significantly impact the experimental outcomes of NSAID toxicity studies. In this study, we examined differences in the susceptibility of in-housebred and commercially purchased mice to indomethacin-induced injury. We further investigated how microbial community structure and function differ between these groups. Our findings advance the mechanistic understanding of the interactions among NSAIDs, the gut microbiome, and host physiology. These results could benefit the reproducibility and translatability of preclinical studies using different mouse sources and shed light on the possible functional role of specific microbial species in modulating NSAID toxicity.

## Materials and methods

### Animals

All the animal experiments were conducted in accordance with the protocols approved by the Institutional Animal Care and Use Committee of the University of North Carolina at Chapel Hill (IACUC approval numbers: 22−170.0 and 21−049.0). The mice were housed in a specific pathogen-free (SPF) facility with a 12-h light/dark cycle and maintained at 20−23 °C with 30%−70% relative humidity. The mice had ad libitum access to drinking water and chow (irradiated Purina PicoLab® Select Rodent 50 IF/6F 5V5R*) for the entire study. In-house bred male and female ‘Tar Heel’ mice were bred at the AAALAC-approved University of North Carolina at Chapel Hill (UNC) animal facility and were originally purchased from the Jackson Laboratory (C57BL/6J). Charles River (C67BL/6N) male and female mice were commercially bred and purchased from Charles River Laboratories. Externally purchased mice were allowed to acclimatize for two weeks at our UNC animal facility prior to further experiments.

### NSAID selection

C57BL/6J mice have a known spontaneous mutation in mitochondrial *Nnt,* which could influence the ability to handle mitochondrial reactive oxygen species known to be generated with the NSAID diclofenac.^20^ To account for this potential discrepancy, we used the NSAID indomethacin, which is known to scavenge superoxide radicals in vitro^21^ and ex vivo in primary human polymorphonuclear leukocytes stimulated with phorbol ester, a potent ROS generator.^22^ Furthermore, C57BL/6J also has a mutation in *Nlrp12,* which could influence neutrophil recruitment, which was examined using two independent methods (described below). Thus, we accounted for strain-specific differences by careful selection of NSAID and specific phenotypic markers to examine microbiota-dependent contributions to indomethacin-induced enteropathy.

### Animal experiment 1: effects of indomethacin on a mouse model in ileum

**Figure. 1.**
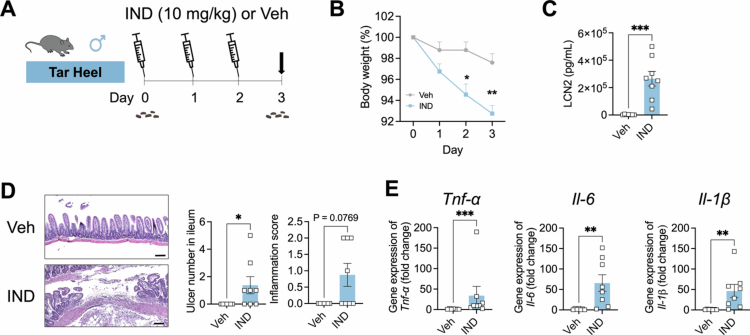
Three-day indomethacin treatment produces gastrointestinal toxicity. A. Scheme of the experimental design, in which in-house bred ‘Tar Heel’ C57BL/6 male mice were treated with indomethacin (IND; 10 mg/kg body weight) or vehicle for 3 d and then euthanized 24 h after the last dose (dark arrow). Fecal material was collected at day 0, as indicated. B. Percent body weight at day 0 significantly decreased in IND-treated mice at days 2 and 3. C. Fecal lipocalin-2 (LCN2) levels at day 3 were significantly higher in IND-treated mice than in control mice. D. Ulcers were found in the ileum of IND-treated mice but not in the ileum of vehicle-treated mice. Representative H&E histological images of the ileum are shown on the left (scale bar = 100 μm), while at right the right, the number of ulcers in the ileum was significantly higher in the IND-treated mice than in the control mice and was not detected in the vehicle-treated animals. E. The expression of the proinflammatory cytokines *Tnf-α, Il−6,* and *Il-1β* normalized by *β-actin* in the ileum was significantly higher in IND-treated mice than in vehicle-treated animals. *n* = 7 in the Veh group; *n* = 8 in the IND group. For the comparisons between treatment groups, statistical significance was determined using two-side t-test. *p < 0.05, **p < 0.01, ***p < 0.001, ****p < 0.0001.

Tar Heel mice (male, 8−10 weeks old) were obtained from the UNC facility and allowed to acclimatize for two weeks prior to study initiation. The mice were randomly assigned to two groups: those that were orally gavaged with indomethacin (dose = 10 mg/kg body weight; *n* = 8) or those that were gavaged with vehicle (Veh; *n* = 7). Gavages were performed once daily in the morning for 3 d (the experimental design is shown in Figure 1A). The vehicle was a sterile solution consisting of 10% dimethylformamide, 0.5% methyl cellulose and 0.5% tween −80. The mice were euthanized 24 h after the final dose of indomethacin or vehicle, and blood and tissues were collected and stored at −80 °C for further analysis. Fecal samples were collected daily and immediately placed on dry ice prior to storage at −80 °C for further analysis.

### Animal experiment 2: effects of indomethacin on in-house bred and commercial bred mice in ileum.

Tar Heel and Charles River mice (male and female, 8−10 weeks old) were obtained and allowed to acclimatize for two weeks prior to study initiation. The tar Heel and Charles River mice were randomly assigned into two groups for both sexes (*n* = 11−19/group/sex): those orally gavaged with indomethacin (10 mg/kg body weight) or vehicle (Veh) for three total days, as described in Animal Experiment 1 (experimental design in Figures S1 and 2 A). The mice were euthanized 24 h after the final dose of indomethacin or vehicle, and blood and tissues were collected and stored at −80 °C for further analysis. Fecal samples were collected daily and immediately placed on dry ice prior to storage at −80 °C for further analysis.

#### Animal experiment 3: comparative metagenomic analysis of gut microbiomes on in-house bred and commercial bred mice.

Tar Heel and Charles River mice (male and female, 8−10 weeks old) were obtained and acclimatized as described above. To investigate the microbial basis for differential indomethacin responses, we performed baseline gut microbiome analysis prior to drug treatment (the experimental design is shown in Figure A). Fresh fecal samples were collected from individual mice, flash-frozen on dry ice, and stored at −80 °C until metagenomic sequencing analysis.

### Measurement of Lipocalin−2 in feces

We quantified intestinal inflammation using the established biomarker fecal lipocalin−2 (LCN−2), a small protein expressed and secreted by epithelial cells, neutrophils and other immune cells.^23^ Fecal samples were thawed, weighed and weight-normalized with PBS containing 0.1% Tween 20, and then vortexed for 10–15 s. The samples were placed on a shaker in a cold room overnight, and the supernatant was collected after centrifugation at 12,000  rpm for 10 min. The content of LCN−2 in the supernatant was determined using a Mouse Lipocalin−2/NGAL DuoSet ELISA Kit (R&D Systems, Catalog #DY1857) according to the manufacturer's instructions. The optical density was determined using a microplate reader (CLARIOstar Plus) at 450 and 540 nm. The wavelength correction was determined by subtracting readings at 540 nm from the readings at 450 nm. The LCN−2 concentration was calculated using a standard curve and expressed as pg/ml per mg of fecal material.

### Reverse-transcriptase-qPCR of inflammatory biomarkers in Ileum

Two centimeters of ileal tissue was obtained from each mouse and frozen at −80 °C with RNAlater (Invitrogen). Total RNA was isolated using RNeasy Mini Kit (Qiagen) according to the manufacturer's instructions. The RNA was reverse transcribed into cDNA using SuperScript III reverse transcriptase (Invitrogen). PCRs (20 µl) were prepared using Applied Biosystems SYBR Green Master Mix (Thermo Fisher Scientific), and qPCR was carried out using a DNA Engine Quantstudio three system (Applied Biosystems). The sequences of the mouse-specific primers (Sigma Aldrich) used to detect inflammatory biomarkers are listed in Table S1**.** The results for the target genes were normalized to *β-actin*using the 2^–ΔΔCT^ method.

### H&E histological staining and ulceration measurement in Ileum

The ileum of each mouse was opened longitudinally, Swiss rolled and fixed in 10% phosphate-buffered formalin (Thermo Fisher Scientific) for 48 h. The tissue sections were routinely processed to paraffin, sectioned at a thickness of 5 μm every 100 μm three times, and stained with hematoxylin and eosin (Epredia Richard-Allan Scientific). The samples were evaluated microscopically (RSS). The samples were assessed using a qualitative scoring system, except for mucosal erosions/ulcerations, which were counted to obtain the total number of erosions/ulcerations in the sample. All three swiss roll samples were evaluated, and mucosal erosions/ulcerations were counted but not duplicated across sections (i.e., three sections had a lesion in the same location, and the lesion was counted only once). The samples were scored according to Table 1.

**Table 1.** Criteria used to score microscopic findings in the ileum.

Finding	1	2	3	4	5
Inflammation (not including regions of ulceration)	Minimal and limited to LP	Mild and limited to LP or minimal but extending into SM	Mild in LP and SM	Moderate to severe in LP & SM or mild + crypt abscesses	Transmural
Distribution of most severe finding	<5%*	6%−30%	31%−60%	>60%	NA
Crypt degeneration and atrophy	minimal	mild	moderate	marked	severe
Distribution of crypt degeneration and atrophy	<5%	6%−30%	31%−60%	>60%	NA

LP = lamina propria of the ileum; SM = submucosa of the ileum; NA = not applicable; *Percent is an estimate.

### PAS-Alcian blue staining and goblet cell quantification

Ileal tissue blocks were prepared from 10% phosphate-buffered formalin-fixed tissues, sectioned (5 μm) and stained with PAS-Alcian blue. Slides were examined and imaged under a light microscope using CellSens software (Olympus). Using ImageJ, the number and area of goblet cells in the ileum were manually quantified by analyzing at least 10 crypt-villus units per mouse.

### Immunohistochemistry

Paraffin-embedded 5 μm tissue sections were deparaffinized, rehydrated, and subjected to heat-induced epitope retrieval (pH 6.0 buffer, Epredia). After blocking endogenous peroxidase (3% H₂O₂, 10 min) and nonspecific binding (10% normal goat serum, 1 h), the sections were incubated with a rabbit anti-Ly6G antibody (Cell Signaling Technology, 1:200) overnight at 4 °C, followed by incubation with a biotinylated goat anti-rabbit IgG (Jackson ImmunoResearch, 1:500, 1 h, RT) and an ABC-HRP complex (Vector, 30 min, RT). The signal was developed with DAB (Epredia), counterstained with hematoxylin (Epredia), and mounted with DPX (Electron Microscopy Sciences). Images were captured using an Olympus IX71 microscope and quantified using ImageJ software. Optical density (OD) was measured to quantify the brown DAB precipitate using the following formula: OD = log (max intensity/mean intensity).

### Detection of indomethacin and its metabolites by LC-MS/MS

Mouse plasma (100 µL) with an internal standard (100 ng/mL) was extracted with 1 mL 80/20 methanol/water and then shaken for 10 min. The extracts were subsequently centrifuged at 10,000 rpm for 10  min, after which the supernatants were transferred and dried down for analysis. The samples were reconstituted in 100 µL of 80/20 methanol/water and analyzed with a Thermo Fisher TSQ Vantage (Thermo Fisher, Bremen, Germany) mass spectrometer coupled to a Waters Acquity Classic UPLC. The samples were introduced via an electrospray source (ESI) at a flow rate of 0.3 mL/min. The electrospray source conditions were set as follows: spray voltage, 3.5 kV; vaporizer temperature, 300 °C; sheath gas (nitrogen), 35 arb; auxiliary gas (nitrogen), 30 arb; and capillary temperature, 350 °C. The scan width was set to 0.700 m/z with a scan time of 0.500 s. The column temperature was set to 45 °C, and the sample temperature was maintained at 10 °C. The samples were analyzed via multiple reaction monitoring (MRM) in positive mode. Separations were conducted on a Waters BEH C18 (2.1 × 100 mm, 1.7 µm) using a flow rate of 0.3 mL/min and the following mobile phases: A- water with 0.1% formic acid and B- acetonitrile with 0.1% formic acid. The gradient was initialized at 90% A and held for 1 min before decreasing to 20% A for 4 min. The gradient then decreased to 0 A for 1 min and was held at 0% A for 2 min. The gradient was then returned to 90% A at 8.10 min and held for 12 min. The injection volume was held at 3 µL for all the samples. Indomethacin and its metabolite indomethacin-acyl-*β*-D-glucuronide were quantified using multiple reaction monitoring in positive ion mode. The data were analyzed with Xcalibur software (ThermoFisher, Bremen, Germany).

### DNA extraction from fecal samples

DNA was extracted from mouse fecal samples using the QIAmp DNA Stool Mini Kit (Qiagen, Valencia, CA) following the manufacturer's instructions, with an additional bead-beating step.^24-26^ The quantity of the extracted DNA was measured using a NanoDrop Spectrophotometer (Thermo Fisher Scientific), and the quality was assessed via 1% agarose gels.

### Whole-genome sequencing and analysis

PCR products were detected via 2% agarose gels via electrophoresis and purified using the Qiagen Gel Extraction Kit (Qiagen, Germany). The sequencing libraries were generated using NEBNext Ultra DNA Library Pre-Kit for Illumina, following the manufacturer's recommendations, and index codes were added. Library quality was assessed using the Qubit 2.0 fluorometer (Thermo Fisher Scientific) and an Agilent Bioanalyzer 2100 system. The library was sequenced on an Illumina platform, and whole-genome reads were generated (10 Gb of raw data). Metagenomic data were processed using MetaPhlAn (v4.06) to create a merged taxonomic relative abundance table.^27^ Alpha diversity was calculated via the Shannon diversity index using MetaPhlAn, and the Kruskal‒Wallis rank sum test was employed to compare alpha diversity between groups. Beta diversity was calculated via the Bray‒Curtis dissimilarity matrix using MetaPhlAn, and PERMANOVA was applied to compare beta diversity between groups using Adonis2. Classical multidimensional scaling was applied to the dissimilarity matrix to create a PCoA plot, which was then colored by groups. Kruskal‒Wallis rank sum tests were employed to compare taxa between groups. All the statistical tests were performed in R.^28^ All the plots were obtained using ggplot2 in R.

### Protein-level analysis of metagenomic data

Three publicly available gut metagenomic protein catalogs were obtained (the MPA4 marker gene database, UHGP−100, and CMMG representative genomes).^27^^,^^29^^,^^30^ The MetaPhlAn marker gene database was first converted from nucleic acid to protein sequences using Prodigal (v2.6.3).^31^ For each protein of interest, the enzyme structures and literature were analyzed to create a rubric using methods described and employed previously (see also supplemental materials).^32-36^ Rubrics were employed to identify protein orthologs in all three databases. The resulting protein IDs were merged with their provided taxonomic annotations to create an integrated database of microbial species known to encode an enzyme of interest. For each protein, the species-level MetaPhlAn results were filtered to retain only those species that encoded the protein, and these results were summed to generate percent abundance values for each sample.

### Data analysis

**Figure 2.**
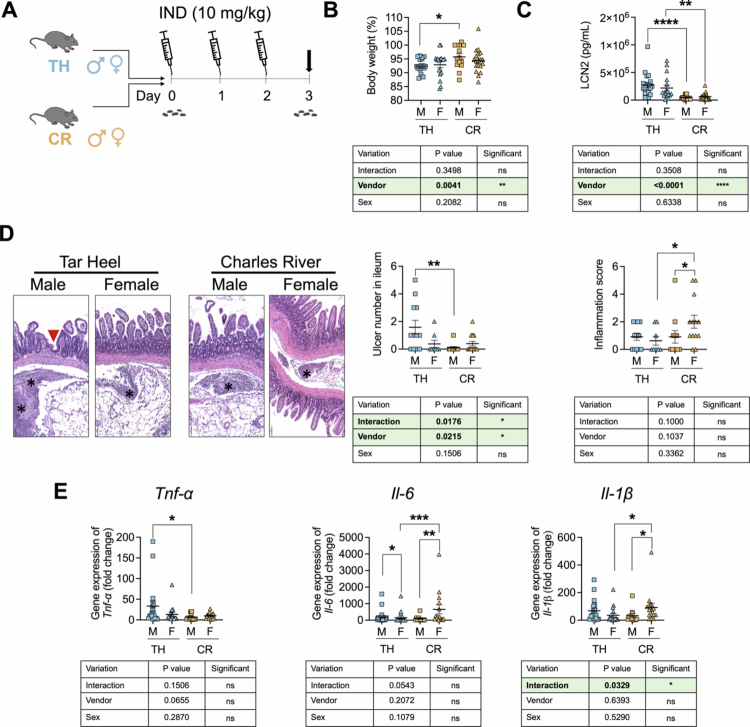
Differential indomethacin-induced GI toxicity in Tar Heel and commercial Charles River mice. A. Scheme of the experimental design, in which in-house bred Tar Heel (TH) and commercially purchased Charles River (CR) C57BL/6 male and female mice were treated with indomethacin (IND; 10 mg/kg body weight) for 3 d and then euthanized 24 h after the last dose (dark arrow). Fecal material was collected at day 0, as indicated. B. Percent of day 0 body weight values show that male (M) CR mice lost significantly less weight than M TH mice did. C. Fecal lipocalin-2 (LCN2) levels at day 3 are significantly higher in M and F TH mice. D. Differential IND-induced ulcerations of the ileum as revealed by representative H&D images. E Histological images of the ileum on the left with an ulcer are indicated with a red triangle, and inflammation is indicated with asterisks (scale bar = 100 μm). On the right, the number of ulcers in the ileum was significantly higher in M TH mice than in control mice, and a significant interaction effect was detected among the groups according to vendor type and sex type via two-way ANOVA. Inflammation scores in the ileum were higher in F CR mice compared to M CR and F TH mice. E. Gene expression of proinflammatory cytokines in the ileum normalized by *β-actin* shows differential effects depending on sex and vendor for *Tnf-α, Il−6,* and *Il-1β*. The GI toxicity between Tar Heel and Charles River mouse experiments was compared via two-way ANOVA according to vendor type and sex type, followed by Tukey–Kramer's method. For the comparisons between two treatment groups, statistical significance was determined using two-side t-test. *p < 0.05, **p < 0.01, ***p < 0.001, ****p < 0.0001. Additional Veh and IND data for both vendors and sexes are shown in Figure S1.

**Figure 3.**
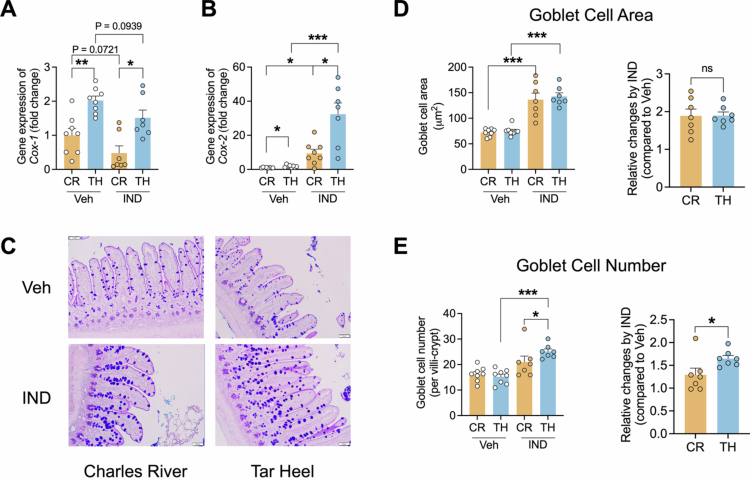
Indomethacin treatment altered cyclooxygenase (COX) expression and goblet cell parameters in the ileum of Tar Heel mice and Charles River mice. A. The gene expression of *Cox−1* in ileum tissue was downregulated in both Charles River and Tar Heel mice treated with indomethacin. B. The gene expression of *Cox−2* in ileum tissue was concurrently upregulated following indomethacin treatment of Charles River and Tar Heel mice. C. Alcian Blue/periodic acid Schiff staining of the ileal mucus in mice revealed goblet cells stained light blue (scale bar = 50 μm). D. Treatment with indomethacin significantly increased the goblet cell area (~1.9 times larger compared to Veh) in mice from both vendors, with no difference observed between the vendors. E. Treatment with indomethacin significantly increased the number of goblet cells in TH mice only, with a significantly higher increase observed in TH mice compared to CR mice. CR: Charles River; TH: Tar Heel. *n* = 7−8/group. Statistical significance was determined using two-side t-test. *p < 0.05, **p < 0.01, ***p < 0.001. ns: no significance.

**Figure 4.**
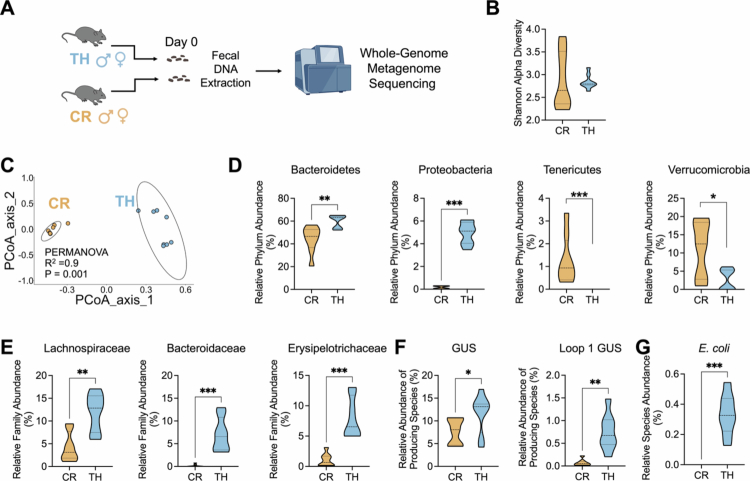
Fecal metagenomic analysis revealed altered *Bacteroidetes*, *Proteobacteria* abundance and *β*-glucuronidase (GUS)-producing species in Tar Heel mice. A. Schematic of the experimental design in which day 0 fecal samples from male and female Charles River and Tar Heel mice were examined via whole-genome metagenome sequencing. B. Alpha diversity did not differ between CR and TH mice. C. Beta diversity significantly differed between CR mice and TH mice. D. At the phylum level, the abundances of Bacteroidetes and Proteobacteria were significantly higher in TH mice, while the abundance of Tenericutes and Verrucomicrobia were significantly higher in CR mice. E. At the family level, the abundances of Lachnospiraceae, Bacteroidaceae and Erysipelotrichaceae were significantly higher in TH mice. F-G. GUS- and loop 1 GUS-producing species, as well as the relative abundance of *Escherichia coli*, were significantly greater in TH mice than in WT mice. CR: Charles River; TH: Tar Heel. *n* = 8/group. Kruskal‒Wallis rank sum tests were used to compare all functional pathways of interest between the vendors. *p < 0.05, **p < 0.01, ***p < 0.001, ****p < 0.0001.

**Figure 5.**
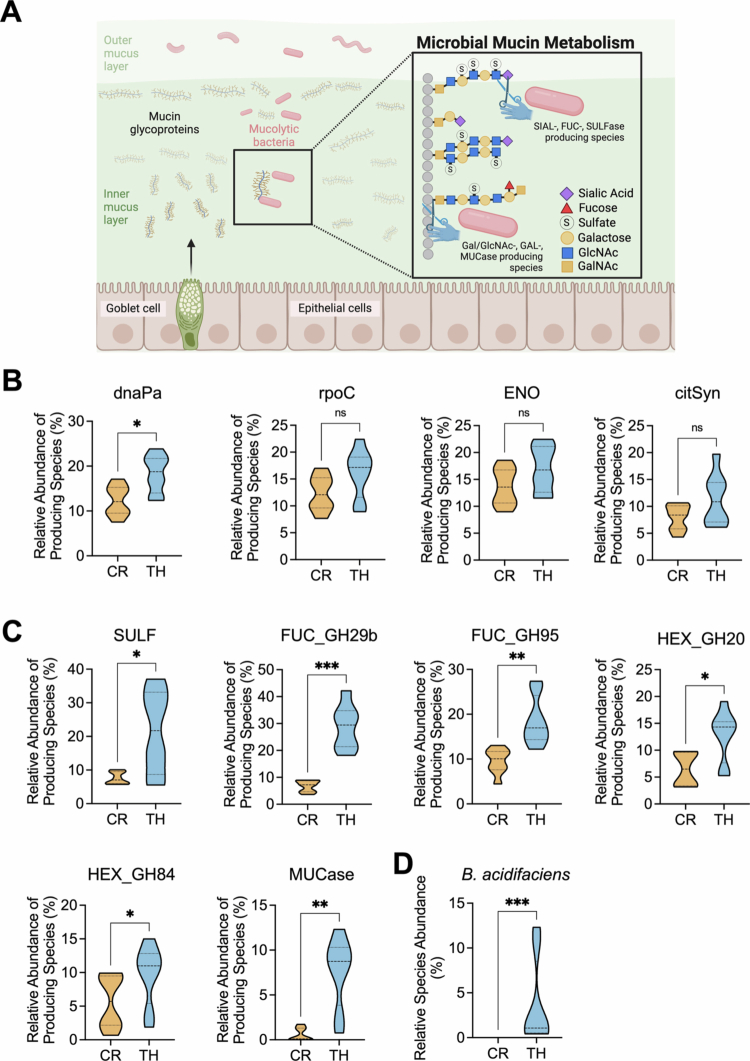
Bacterial species encoding mucolytic enzymes showed increased abundance in Tar Heel mice. A. Schematic of intestinal mucin polysaccharides showing sites of activity of microbial sulfatases (SULFs), fucosidases (FUCs), hexaminidases (HEXs, including *N*-acetyl-glucosidases and *N*-acetyl-galactosidases), and mucinases (MUCase). (Figure created with BioRender.com) Created in BioRender. Sekela, J. (2025) https://BioRender.com/v7qwt9x. B. The relative abundances of gut microbial species encoding ‘control’ microbial enzymes, such as the RNA polymerase rpoC gene, the enolase (ENO) gene of glycolysis, and the citrate synthase (citSyn) gene of the citric acid cycle, did not differ between the TH and CR mice, whereas the TH mice presented increased abundance of the DNA polymerase gene dnaPa. C. TH mice presented relatively high relative abundances of gut microbial species encoding mucin polysaccharide-degrading sulfatase (SULF), fucosidase (FUC, both family GH29b and GH95 factors), hexosaminidase (HEX, both family GH20 *N*-acetyl-galactosaminidase and GH84 *N*-acetyl-glucosaminidase factors) and mucinase (MUCase) enzymes. D. The relative species abundance of the mucolytic bacteria *Bacteroides acidifaciens* was significantly higher in TH mice than in control mice. CR: Charles River; TH: Tar Heel. *n* = 8/group. Kruskal‒Wallis rank sum tests were used to compare all functional pathways of interest between the vendors. *p < 0.05, **p < 0.01, ***p < 0.001, ****p < 0.0001. ns: no significance.

Processed data are expressed as the mean ± standard error of the mean (SEM). For the comparison between treatment groups, statistical significance was determined using a two-sided t test (Mann‒Whitney test) to analyze unpaired data (Figure 1). Analysis of intestinal toxicity in male and female mice (Figure S1) was performed via two-way ANOVA according to sex and treatment following Tukey‒Kramer's method.^37^ For comparisons between groups, statistical significance was determined using two-side t-tests (Figure S1). Comparisons of GI toxicity between groups (Figure 2) were performed via two-way ANOVA following Tukey‒Kramer's method. For the comparisons between treatment groups, statistical significance was determined using a two-sided t test (Mann‒Whitney test) to analyze unpaired data (Figure 3 and Figure S3–S4). Kruskal‒Wallis rank sum tests were used to compare proteins of interest in the metagenomic data between groups (Figures 4–5). *P* values less than 0.05 are reported as statistically significant. All figures were generated using GraphPad Prism 10 (GraphPad Software) and R software.

## Results

### Indomethacin induces ileal toxicity in mice

To develop a reproducible model of NSAID-induced ileal toxicity, we treated male ‘Tar Heel’ mice bred in-house at the University of North Carolina at the Chapel Hill animal facility with indomethacin. Specifically, the mice were treated with indomethacin (IND; *n* = 8) by oral gavage at a dose of 10 mg/kg body weight or with vehicle (Veh; *n* = 7) for three days and euthanized 24 h after the final dose (Figure 1A). Compared with the mice treated with Veh, the mice treated with IND presented significantly lower body weights by days 2 and 3 (Figure 1B). Compared with Veh-treated mice, IND-treated mice also presented significantly increased fecal lipocalin-2 levels at the end of the study (Figure 1C). The ileal tissues of the IND-treated mice presented increased mucosal erosion and ulceration (Figure 1D), and upregulated the expression of the proinflammatory cytokines *Tnf-α, Il−6,* and *Il-1β* (Figure 1E). Taken together, these results indicate that 10 mg/kg IND delivered by oral gavage for three days produces evidence of ileal toxicity in male Tar Heel mice.

### Differential responses to indomethacin in tar heel and charles river mice

To better understand the factors that may influence NSAID-mediated intestinal injury, we next directly compared IND-treated Tar Heel and Charles River mice of both sexes (Figure 2A). We found that male Tar Heel mice lost significantly more weight than male Charles River mice did (Figure 2B) and that all Tar Heel mice presented significantly higher fecal lipocalin−2 levels than their Charles River counterparts did (Figure 2C). Using immunohistochemical analyses, we found that indomethacin elicits neutrophil recruitment in the intestinal mucosa of both substrains, with Tar Heel-treated mice having a statistically significant increase in neutrophil recruitment relative to vehicle-treated mice (Figure S3A-B). This observation suggests that C57BL/6J mice do not have impaired neutrophil recruitment following indomethacin exposure, despite their known mutation in *Nlrp12*^38^. Compared with male Charles River model mice, male Tar Heel model mice presented significantly more ileal ulcers; conversely, female Charles River model mice presented higher histologic inflammation scores than male Charles River model mice and female Tar Heel model mice did (Figure 2D, Figure S2). Finally, Tar Heel male mice also presented higher ileal *Tnf-α* expression compared to male Charles River mice did, as well as higher *Il−6* expression compared to their female counterparts did. Intriguingly, Charles River female mice presented *Il−6* expression similar to that of male Tar Heel mice, which was significantly higher than that of their male counterparts and female Tar Heel mice. This was also the case for *Il-1β* expression (Figure 2E). Taken together, these data demonstrate that mice from different breeding facilities exhibit differential toxicity and ileal damage in response to oral IND treatment. Specifically, Tar Heel mice appear to be more susceptible to IND-induced ileal ulceration than Charles River mice are. A complete analysis including all vehicle groups is shown in Figure S1, which further supports these findings.

NSAIDs are known to inhibit cyclooxygenase enzymes; therefore, we next quantified the ileal gene expression of *Cox−1* and *Cox−2,* which synthesize prostaglandins from arachidonic acid. We found that indomethacin inhibited *Cox−1* gene expression in both Charles River and Tar Heel mice (Figure 3A). COX−1 inhibition by indomethacin is known to upregulate COX−2 expression,^39^ which occurs in both substrains, although the magnitude of *Cox−2* induction is significantly higher in Tar Heel compared to Charles River mice (Figure 3B). Prostaglandins synthesized by COX−1 and COX−2 play important roles in regulating mucus secretion from goblet cells;^15^ Therefore, we next examined the ileal goblet cells of indomethacin-treated mice using PAS-Alcian blue staining (Figure 3C). Compared to Veh group, IND treatment significantly increased the ileal goblet cell area in both the Charles River and Tar Heel groups (Figure 3D). Moreover, IND also increased the number of ileal goblet cells in Tar Heel mice but not in Charles River mice (Figure 3E); this may be due to the higher *Cox−2* expression observed, which is expected to result in a higher level of prostaglandin production. Overall, there were no differences in goblet cell size between Charles River and Tarheel mice treated with IND; however, IND induced greater numbers of goblet cells in Tarheel mice compared to their Charles River counterparts did (Figure 3C–E). These findings further highlight the differential response to IND in these groups.

### Tar heel and charles river mice have distinct intestinal microbiomes and *β*-glucuronidase producing taxa

Given that Tar Heel and Charles River mice display differential responses to IND, we hypothesized that their gut microbiomes may differ prior to NSAID treatment. To test this hypothesis, we performed whole-genome metagenome sequencing on fecal samples obtained at the start of the experiment prior to vehicle or IND exposure (Figure 4A). However, the alpha diversity did not differ between the groups (Figure 4B), beta diversity was significantly different between the Tar Heel and Charles River cohorts (Figure 4C). Taxonomically, at the phylum level, we found that Tar Heel mice contained significantly higher abundances of *Bacteroidetes* and *Proteobacteria*compared to Charles River mice, while the Charles River animals contained more *Tenericutes* and *Verrucomicrobia* (Figure 4D, Table S2). These differences also extended to the family level, where *Lachnospiraceae*, *Bacteroidaceae* and *Erysipelotrichaceae* were significantly more abundant in the Tar Heel cohort compared to Charles River mouse cohort (Figure 4E, Table S3).

NSAIDs such as IND are known to reach the gut as inactive glucuronides and are reactivated by gut microbial *β*-glucuronidase (GUS) enzymes.^40^ We previously demonstrated the importance of *β*-glucuronidases in driving NSAID-induced enteropathy^10^^,^^11^ and identified the specific molecular features of GUS responsible for carrying out this declucuronidation chemistry.^40^ Specifically, we found that Loop 1 GUS are the primary class that reactivates NSAID glucuronides in vitro and in vivo. Thus, we also examined the composition of gut microbial taxa that encode GUSs, including all GUS isoforms, as well as specific structural classes, including Loop 1, associated with efficient drug-glucuronide processing. We found that Tar Heel mice presented significantly higher abundances of bacterial species encoding genes for all GUS enzymes and specifically for Loop 1 GUS proteins (Figure 4F, Table S4). Moreover, we found that *E. coli,* a loop 1 GUS producer, was significantly more abundant in Tar Heel compared to Charles River mice (Figure 4G). This finding is consistent with our previous findings that loop 1 GUS are the predominant NSAID-glucuronide-reactivating GUSs.^40^ Accordingly, we found that Tar Heel mice have higher (albeit not statistically significant) levels of indomethacin and indomethacin glucuronide in plasma (Figure S4). Taken together, these findings indicate that the Tar Heel and Charles River mice in our study presented baseline compositional and functional differences in their gut microbiomes. Accordingly, poor outcomes were observed in Tar Heel mice treated with indomethacin (body weight, fecal lipocalin-2, and ulcers; Figure 2) may have arisen from the distinct functional capacities present in the fecal microbiomes of this cohort.

### Higher metagenomic abundance of taxa encoding mucolytic enzymes in Tar heel mice

In addition to GUS activity, we also examined other gut microbial functions that could contribute to differences between Tar Heel and Charles River mice in susceptibility to IND. Given the differences noted in goblet cell numbers in these cohorts after IND treatment, we next explored the abundances of gut microbial species that encode enzymes capable of degrading mucin polysaccharides and the mucin backbone (Figure 5A). Specifically, we examined the abundances of gut microbial taxa encoding sulfatases, fucosidases, sialidases, *N*-acetylgalactosaminidases, *N*-acetylglucosaminidases, and mucinases in the fecal metagenomics data collected from our mouse cohorts. These enzyme families were chosen based on those expected to be involved in the microbial breakdown of intestinal mucus (Figure 5A). We also chose four ‘control’ enzymes expected to be present in microbes regardless of mucin metabolism: DNA polymerase (dnaPa), RNA polymerase (rpoC), enolase from glycolysis (ENO), and citrate synthase from the TCA cycle (citSyn).

We found no significant differences in the abundances of microbial taxa that encode three of the four control enzymes in Tar Heel and Charles River mice (Figure 5B). In contrast, we found that the abundances of fecal microbes encoding the following mucolytic enzymes were significantly higher in Tar Heel mice: sulfatase (SULF), fucosidases from the glycoside hydrolase (GH) families 29 and 95 (FUC_GH29, FUC_GH95), the hexosaminidase HEX) proteins *N*-acetylglucosaminidase (HEX_GH20) and *N*-acetylgalactosaminidase (HEX_GH84) and the mucinase (MUCase) enzyme (Figure 5C, Table S4). Thus, the fecal microbiomes of the Tar Heel mice in our study contained a higher abundance of microbes encoding these mucolytic factors than did those of the Charles River mice. Finally, we found that the mucolytic species *B. acidifaciens*, which encodes each of the mucus-degrading factors outlined above, was also significantly more abundant in Tar Heel than in Charles River mice (Figure 5D). Collectively, these results demonstrate differential abundances of specific bacterial species encoding mucolytic enzymes between Tar Heel and Charles River mice.

## Discussion

In this study, we discovered significant differences in indomethacin-induced toxicity and ileal damage between mice from different facilities, either bred in-house (Tar Heel) or purchased from Charles River. Compared with Charles River mice, Tar Heel mice presented increased ileal toxicity, as demonstrated by significantly greater body weight loss, increased ulcer numbers, and elevated fecal lipocalin-2 (LCN-2) levels. Additionally, Tar Heel mice presented a significantly higher number of goblet cells following indomethacin exposure. To investigate the mechanistic basis for these differential drug responses, we analyzed the baseline gut microbiota of both mouse populations. Whole-genome metagenomic sequencing of fecal samples revealed distinct intestinal microbiomes between Tar Heel and Charles River mice, particularly in terms of the abundance of *β*-glucuronidase-producing taxa. Additional analysis also revealed increased metagenomic abundance of mucolytic enzyme-encoding taxa in Tar Heel mice. Our findings suggest that differences in intestinal microbiomes – specifically in *β*-glucuronidase-producing and mucolytic enzyme-producing bacteria – may contribute to the distinct intestinal toxicity responses to indomethacin between mouse populations.

The C57BL/6 mouse background is widely used to study the role of microbes in various disease models; however, differences between mouse vendors, genetic lineages and husbandry protocols have been shown to contribute to variations in phenotypes and to nonreproducibility of experimental results. A large study of the gut microbial composition by 16S rRNA sequencing of C57BL/6 mice from three different vendors (The Jackson Laboratory, Charles River Laboratories, and Taconic Biosciences) in the United States revealed considerable variation across eight sites, highlighting the importance of environmental conditions on microbial dynamics, which may contribute to experimental reproducibility.^18^ It was found that the microbiomes of Charles River mice were more diverse than those of other vendors, while the microbiomes of Jackson Laboratory mice changed less with time in basal mice.^18^ Our whole-genome metagenomic analysis revealed fundamental differences in baseline gut microbiomes between Tar Heel and Charles River mice prior to indomethacin exposure, providing crucial insights into their differential drug responses. Distinct differences were observed in beta diversity and specific taxonomic compositions. Tar Heel mice presented significantly higher abundances of *Bacteroidetes* and *Proteobacteria*, along with *Lachnospiraceae*, *Bacteroidaceae*, and *Erysipelotrichaceae*. An increased prevalence of *Proteobacteria* is a potential diagnostic signature of dysbiosis and risk of disease.^41^ These baseline microbiome differences suggest inherent variations in the metabolic capabilities of the gut microbiota between the two mouse populations, which could significantly influence their response to xenobiotics.

LCN−2 is an essential antimicrobial component of the innate immune system released by epithelial and immune cells, chief among them being mature neutrophils; LCN−2 is an established biomarker of intestinal mucosal damage and inflammation.^23^^,^^42^^,^^43^ A previous study showed that C57BL/6 male mice (purchased from Janvier, Le Genest St Isle, France) treated orally with indomethacin at a dose of 0.25 mg/mouse (~10 mg/kg body weight) for 5 d presented significantly increased fecal LCN−2 levels.^44^ In addition, we found that oral indomethacin administered at 10 mg/kg body weight for 3 d significantly increased fecal LCN−2 compared to vehicle group (Figure 1C). Moreover, we detected distinct responses between Tar Heel and Charles River mice after treatment with indomethacin. Tar Heel mice presented significantly greater intestinal vulnerability, characterized by elevated fecal LCN−2 levels and increased ileal ulcer numbers (Figure 3E). In contrast, indomethacin exposure elicited similar increases in the gene expression of proinflammatory cytokines in mice sourced from either vendor. This suggests that the differences in susceptibility to NSAID-induced enteropathy between the vendors could arise from mechanisms beyond inflammatory gene regulation, such as the microbiome or/and mucosal integrity factors.

By inhibiting cyclooxygenase enzymes, indomethacin reduces the production of prostaglandins, which normally protect the intestinal mucosa. Consistent with previous reports, in both Charles River and Tar Heel mice, indomethacin reduced the gene expression of the constitutive enzyme *Cox−1* while increasing the inducible gene expression of *Cox−2*^39^ (Figure 3A–B). Goblet cells play a crucial role in maintaining intestinal barrier function through mucus production, and their hyperplasia is often observed as a protective mechanism against intestinal injury caused by different factors, including NSAIDs.^14^ This adaptive, compensatory hyperplasia response is aimed primarily at enhancing mucus production to recreate the protective barrier in the intestinal epithelium. In our study, we found that there was a differential goblet cell response to indomethacin between the vendors. While both Tar Heel and Charles River mice presented increased goblet cell size following indomethacin exposure, Tar Heel mice presented a significantly higher number of goblet cells. This vendor-specific difference in goblet cell hyperplasia suggests distinct host-protective responses to NSAID-induced injury. A previous study reported that, compared with control treatment, treatment with indomethacin (10 mg/kg) could significantly increase the goblet cell area in the small intestine of C57BL/6J mice.^15^ The enhanced goblet cell response in Tar Heel mice may be an attempt to compensate for the increased intestinal damage they experience with indomethacin.

A key mechanism underlying NSAID-induced intestinal toxicity involves the reactivation of drug conjugates by bacterial GUS enzymes in the gut.^10^^,^^11^ Our metagenomic analysis revealed significant differences in the abundance of GUS-producing bacteria between vendors, particularly in species expressing Loop−1 GUS enzymes, which are known to be highly efficient in drug conjugate processing.^40^ Notably, the significantly higher abundance of *E. coli*, a known loop−1 GUS producer, in Tar Heel mice may result in enhanced local reconversion of nontoxic indomethacin glucuronide conjugates to active indomethacin, contributing to increased intestinal toxicity. In concordance, we found that circulating levels of indomethacin-glucuronide were lower in Tar Heel mice than in Charles River mice (Figure S4). This enhanced capacity for drug reactivation provides a possible mechanistic explanation for the increased susceptibility to indomethacin-induced toxicity observed in Tar Heel mice. These findings align with previous studies demonstrating the critical role of bacterial GUS in NSAID enteropathy^10^^,^^11^^,^^45^ and highlight how vendor-specific differences in microbiome composition, particularly in the abundance of bacteria capable of drug metabolism, can significantly impact experimental outcomes in pharmacological studies.

Another mechanism that may enhance NSAID-induced intestinal toxicity is an increase in microbial mucolytic enzymes, which are strongly linked to IBD pathophysiology and characterized by damage to mucosal integrity and mucus barrier dysfunction. Microbial mucolytic enzymes can break down mucus by degrading its structural components, primarily mucins.^13^^,^^46^ These enzymes play a significant role in the interaction between microbes and host mucus layers. There is evidence suggesting that IBD patients have increased levels of certain mucolytic bacteria (e.g., *Ruminococcus gnavus* and *R. torques*), contributing to mucus barrier dysfunction and the exacerbation of disease symptoms.^47^ Animal models provide further insights into the mechanisms of mucolytic enzyme-induced intestinal damage. Indomethacin treatment significantly increased the abundance of the mucolytic enzyme-producing taxa *Akkermansia muciniphila, Enterococcus spp.,* and *Clostridium cluster* XIVa in C57BL/6 male mice (purchased from Janvier, Le Genest St Isle, France).^44^ Yoshihara et al., showed that direct administration of *A. muciniphila* caused thinning of the jejunal mucus layer.^48^ In our study, we observed a significantly higher abundance of the mucolytic enzyme-encoding species *B. acidifaciens* in Tar Heel mice at baseline, which may create vulnerability in the intestinal barrier due to increased mucus degradation. We propose a mechanism whereby enhanced microbial mucolytic activity toward the intestinal mucus enhances the vulnerability of the mucosa to NSAID-induced injury, resulting in an exaggerated response to NSAID exposure. To date, no studies have compared the abundance of mucolytic enzyme-encoding species before NSAID treatment. It is possible that the relative abundance of mucus-degrading species could be a predictive biomarker for NSAID-induced intestinal damage. This may be leveraged for the clinical pain management of individuals with inflammatory bowel diseases, for whom NSAIDs are known to induce disease relapse/flares.^49^

In addition to their distinct gut microbiome profiles, the C57BL/6 mice in this study represent two substrains – C57BL/6N (Charles River) and C57BL/6J (Tar Heel) – which may independently contribute to the differential indomethacin response. Both strains have been previously sequenced, and a number of indels and SNPs that contribute to phenotypic differences of varying penetrance have been reported.^50^ Some of these could potentially influence responses to NSAIDs. For example, C57BL/6J mice have a mutation in the *Nlrp12* gene, resulting in impaired neutrophil recruitment following bacterial infection.^38^ Intriguingly, we detected increased fecal levels of the neutrophil product LCN−2 in C57BL/6J Tar Heel mice. These findings were somewhat unexpected given their known *Nlrp12* mutation. However, we did not observe impaired neutrophil recruitment in Tar Heel mice, as assessed by immunohistochemical analysis of ileal sections stained with the neutrophil marker Ly6G (Figure S3A–B). These findings suggest that host factors such as neutrophil recruitment are less likely to be key drivers of indomethacin-induced enteropathy. Further investigation is needed to understand how specific C57BL/6 substrain differences contribute to responses to NSAIDs and other medications.

Several clinical reports implicate female sex as a risk factor for clinically relevant gastrointestinal adverse effects of a variety of medications, including NSAIDs. However, many of these studies are confounded by a major issue: females often receive more medication than males do because adult dosages are rarely adjusted based on body weight. Moreover, there are complex sex differences in pharmacokinetics and pharmacodynamics between males and females. We observed a higher number of indomethacin-induced intestinal ulcerations in male Tar Heel mice compared to male Charles River mice, but this difference was not observed in female mice. This suggests that an interaction between vendor and sex may influence NSAID susceptibility. Future work is planned to further understand this phenomenon.

In conclusion, our study shows marked vendor-specific variations in NSAID-induced intestinal toxicity between mice from different breeding facilities, demonstrating the profound complexity of host‒microbiome interactions in drug-related pathophysiology. Our comparative analysis of Tar Heel and Charles River mice revealed significant disparities in susceptibility to indomethacin-induced intestinal damage, characterized by marked differences in weight loss, ulcer severity, fecal LCN2 levels, and ileal goblet cell morphology. Notably, despite similar inflammatory cytokine gene expression, the substantial variations in drug response suggest that the fundamental differences may lie in the bacterial microenvironment and its intricate interactions with the intestinal epithelium. Metagenomic analysis revealed two pivotal mechanisms that may underlie these distinct drug responses: first, variations in *β*-glucuronidase-producing bacteria potentially modulating drug metabolism; second, differences in mucolytic enzyme-producing bacteria that may compromise mucosal integrity. Ultimately, several factors, including both microbiome and host differences, likely impact the susceptibility of mice to NSAID-induced injury. These findings not only underscore the critical importance of vendor source standardization in preclinical research but also highlight the emerging paradigm of microbiome-mediated drug responses.

## Supplementary Material

Supplementary material Vendors 10X3_Supplementary inflammation_revision 0825.

## Data Availability

The authors confirm that the data supporting the findings of this study are available within the article and its supplementary materials. Metagenomic sequencing data are deposited in the National Library of Medicine Sequence Read Archive with the unique identifier PRJNA1246137.
